# On the Use of Morphia in Uræmia
^1^A paper read before the Bath and Bristol Branch of the British Medical Association.


**Published:** 1902-09

**Authors:** T. M. Carter, F. H. Edgeworth

**Affiliations:** Assistant-Physician to the Bristol Royal Infirmary; Assistant-Physician to the Bristol Royal Infirmary


### ON THE USE OF MORPHIA IN URÆMIA.[1]

BY

T. M. CARTER, M.R.C.S., L.R.C.P., and F. H. EDGEWORTH, M.B., B. Sc., B. A.,

*Assistant-Physician to the Bristol Royal Infirmary.*

---

THERE is a curious and interesting divergence of opinion as to the value of morphia in the treatment of uræmia, to which we wish to direct your attention. On the one hand Osler,[2] after saying that uræmic convulsions should be treated by inhalation of chloroform, bleeding, and diaphoresis by a hot-air bath, proceeds as follows:—"For the restlessness and delirium morphia is indispensable. I have never seen any ill-effects or any tendency to coma. It is of special value in dyspnœa and Cheyne-Stokes breathing of advanced arterio-sclerosis with chronic uræmia." On the other hand, Dickinson,[3] who may be taken as representing the traditional English view, says: "Opium and its derivatives should be avoided in the convulsive and every other stage of organic albuminuria, save only with the lardaceous kidney, when they are permissible and sometimes useful, but not when the condition is productive of convulsion or any other uræmic symptom."

These views are as divergent as it is possible for them to be —Dickinson advises us to altogether refrain from the use of a drug in uræmia, which Osler says is indispensable and has never in his experience produced any ill results. It may be noted that Dickinson does not give any reasons for the faith which is in him: for instance, he does not say that he has seen ill effects from the use of morphia in uræmia.

The opinion of younger English physicians is a little more favourable. In the *Practitioner*[4] for last year are some valuable articles on Bright's disease, and that on treatment is written by

[1] A paper read before the Bath and Bristol Branch of the British Medical Association.

[2] *The Principles and Practice of Medicine.*

[3] Allbutt's *System of Medicine*, vol. iv., 1897.

[4] 1901, lxvii. 499 *et seq.*

Hale White,[1] who, though not mentioning the use of morphia in the general treatment of uræmia, or in that of uræmic headache, sleepnessness or dyspnœa, yet in the paragraph on uræmic convulsions says. "Perhaps, if bleeding and other methods of treatment have done no good, the best drug is morphine, although, as these patients have diseased kidneys, it must be given with the greatest care."

Curiously enough, in the same number of the *Practitioner* Dr. Berry Hart strongly recommends the employment of morphia in puerperal eclampsia—a condition which, though not identical with, is yet closely allied to uræmia.

Modern English opinion then hesitates between the two extremes: though it does not absolutely condemn like Dickinson, it does not strongly recommend as does Osler.

In view of these divergences of opinion, we would wish to bring before your notice a case of granular kidney and chronic uræmia where morphia was used for some two years, with the happiest results.

In the summer of 1899 a man, aged 60 years, fat and heavily built, came under the care of one of us with symptoms and signs of chronic renal disease. He was anæmic, with a high-tension pulse—80 to the minute—and thickened arteries. The left ventricle of the heart was enlarged, and there was a markedly accentuated aortic second sound, with a prolonged booming first sound. He was a little dyspnœic on slight exertion, but there were no physical signs of heart failure. The lungs and abdomen were normal. The urine was constantly albuminous, of low specific gravity, and from 60 to 80 ozs. were passed daily. There was no albumiuric retinitis. He was given iron, saline purgatives, erythrol tetranitrate as a vaso-dilator, and was advised to take Turkish baths.

During the autumn of 1899 he had several attacks of very high vascular tension, producing temporary mitral regurgitation.

On November 27th the patient was seized with uræmic coma and convulsions. When seen he was unconscious, breathing stertorously, slightly livid, with occasional clonic convulsions of the face and both arms. The pupils were equal,

[1] *Practitioner*, 1901, lxvii. 658.

slightly dilated, and there was a slight corneal reflex in both eyes. The pulse was 80 per minute, regular, full, and of low tension. This condition had come on suddenly two hours previously, and had persisted unchanged. The patient was bled to 20 ozs. The convulsions ceased a little after the blood had begun to flow, and by the end of the venesection he recovered consciousness. He slept soundly that night, and on the following day he showed no signs of uræmia or of paralysis.

On the 29th, at 4 a.m., he became delirious, and remained in that condition all day in spite of 20 grains of bromide every other hour. A vapour bath, from 9 to 9.30 p m., produced satisfactory diaphoresis, but violence continued, and at 9.45 p.m. he was given $\frac{1}{4}$ gr. morphia sulphate hypodermically. The delirium continued to be so violent that it was necessary to keep two men with him until 1 a.m., when another $\frac{1}{4}$ gr. of morphia was given. He soon became drowsy and passed into restless sleep. The morphia produced very satisfactory sweating. At 5 a.m. he awoke and gradually became more and more noisy. A third $\frac{1}{4}$ gr. was given. He quickly fell asleep, slept for two hours, and awoke less delirious and anxious to take milk. He was slightly delirious all that day, and was given one more dose of morphia in the evening. The following morning all trace of delirium had disappeared. He gradually grew stronger, and was able to resume work by the middle of January, 1900.

Subsequently to this initial attack, definite attacks of some phase or other of uræmia occurred on the following dates:—On February 1st, 1900, persistent vomiting took place, continuing all through the day and following night. It was stopped by the injection of $\frac{1}{4}$ gr. of morphia on the morning of the 2nd. Intense headache in the following week was relieved by liq. morph. mur. ♏ iij. t.d.s., and his general condition improved so much that the patient was able to get up and do a little work after the 10th. Small doses of morphia and potassium iodide were continued during the remainder of that month.

On May 9th the patient had two attacks of uræmic convulsions and unconsciousness at half an hour's interval.

Quarter of a grain of morphia was given. Sound sleep and profuse diaphoresis followed the injection, and four hours later he was able to be put to bed. In two days he was so well that he got up and resumed work, but continued to take morphia and pot. iod. for about a fortnight.

On July 3rd there was a similar attack, which was similarly treated. All effects passed off in five days.

On October 1st the patient had a severe attack of vomiting, headache and giddiness. Quarter of a grain of morphia hypodermically, followed by ♏ v. liq. morph. mur. 4^is horis, with purging by salines, enabled him to be about again in four days.

On January 13th, 1901, muscular twitchings and nausea yielded quickly to liq. morph. mur. ♏ v. t.d.s. by mouth, with free purging.

On February 16th severe dyspnœa and vomiting, with great feeling of depression and general illness, were relieved by injections of morphia on the 16th and 18th. He was able to resume work on the 20th.

On April 20th the patient had a slight uræmic convulsion without loss of consciousness. One injection of morphia, followed by liq. morph. mur. ♏ iij. with pot. iod. gr. v. t.d.s. for a week, enabled him again to resume work. His general health, however, was becoming enfeebled, and his work, noticeably to all his friends though not to himself, was much deteriorating, apparently through defective vision.

In June he began to complain of defective sight, and suffered greatly from mental depression. Mr. Cross, whom he consulted, reported that albuminuric retinitis was present in both eyes.

On August 17th the patient became delirious, with loss of memory and some delusions. On September 20th he had an attack of cerebral hemorrhage, and died two months later. No *post-mortem* examination was permitted.

The patient was fairly careful as to his diet throughout the course of his illness, and abstained entirely from alcohol after his first uræmic convulsion in 1899. He regularly obtained a loose action of the bowels by taking a morning dose of saline, and always passed from 60 to 80 ozs. of clear, pale urine, of sp. gr. 1005—1010, in which there was usually a trace of albumin.

On reviewing the patient's illness it is seen that during nearly two years he was liable to uræmic attacks—in the varied forms of convulsions, coma, headache, vomiting, dyspnœa, and delirium—phenomena which yielded on each occasion to the administration of morphia. It is further noticeable that the drug produced diaphoresis, and did not diminish the quantity of urine passed. At no time in the course of its frequent use did it give rise to any untoward symptom or occasion any anxiety.

Such a history as this casts much doubt on the correctness of Dickinson's advice, at any rate in cases of chronic Bright's disease, and lends strong support to the truth of Osler's statements.

